# Comparison of automated bone scan indexes in patients with prostate cancer: aBSI and BONENAVI

**DOI:** 10.1007/s12149-026-02244-7

**Published:** 2026-06-09

**Authors:** Takeshi Matsumura, Kenichi Nakajima, Atsushi Mizokami, Hiroshi Wakabayashi, Jens Richter, Seigo Kinuya

**Affiliations:** 1 Department of Nuclear Medicine, Kanazawa University, 13-1 Takara-Machi, Kanazawa, 920-8640 Japan; 2 Department of Urology, Kanazawa University Graduate School of Medical Sciences, Kanazawa, Japan; 3 EXINI Diagnostics, Lund, AB Sweden

**Keywords:** Bone scan index, Metastasis, Software, Quantification

## Abstract

**Objective:**

Bone scan indexes (BSIs) are frequently applied in Japan, Europe and the USA. The BONENAVI (Japan) and aBSI (Europe and USA) software programs were developed based on different training and validation datasets to calculate BSIs. However, whether they are compatible has not been systematically determined. We compared BSIs generated by these programs in a patient cohort to clarify correlations between them.

**Methods:**

We retrospectively analyzed 339 scintigraphic data from 34 patients (mean age, 69.9 ± 9.1 [48–89] y) with prostate cancer who underwent whole-body bone scintigraphy to evaluate metastatic bone disease. Anterior and posterior whole-body scans with ^99m^Tc-methylene-diphosphonate (MDP) were processed then BSIs were computed by aBSI and BONENAVI. Systematic differences between aBSI and BONENAVI were evaluated using Pearson’s correlation coefficients and Bland–Altman analysis. We also categorized BSIs during follow-up as decreased, unchanged, or increased, and evaluated agreement between longitudinal changes.

**Results:**

The mean number of bone scans per patient was 10.0 ± 6.8 (1−26), and longitudinal changes in BSIs were evaluated using 305 pairs of follow-up studies. The automatic BSIs from aBSI and BONENAVI were 3.23% ± 3.47% and 2.91% ± 2.84%, respectively. Both strongly correlated (*r* = 0.947, *p* < 0.001). Bland–Altman plots revealed that aBSI tended to yield slightly higher BSIs (mean difference, + 0.31% (95% CI, 0.19−0.44, *p* < 0.001). A subgroup analysis of BSIs > 7% revealed higher automatic BSIs from aBSI than BONENAVI (mean difference, + 1.57%; 95% CI, 0.99−2.15; *p* < 0.001). The rate of longitudinal changes in BSI during follow-up substantially agreed (78.7% vs. 76.4% with respective thresholds of ± 0.5% and ± 0.3% (κ = 0.63, Cramer’s V = 0.65, *p* < 0.0001 for both).

**Conclusion:**

The aBSI and BONENAVI BSIs computed by the software programs strongly correlated and longitudinal changes during follow-up studies generally concurred. However, BSIs from aBSI tended to be higher among patients with an increased disease burden compared with BONENAVI. Clinicians and investigators should be aware of these differences when interpreting or comparing results among software programs.

## Introduction

The bone scan index (BSI) is a quantitative imaging biomarker that expresses the extent to which metastatic tumors have spread to the skeleton as a ratio (%) of the total skeletal mass. The concept was originally introduced in 1998, when BSIs derived from planar whole-body bone scintigraphic images strongly correlated with tumor burden and the prognosis of metastatic prostate cancer [1]. Early BSI implementation relied on manual or semi-automated interpretation, which limited its clinical applicability due to the need for specialized expertise and timely analyses.

The clinical application of BSI started to advance along with the development of automated image analysis software based on artificial intelligence or artificial neural-networks (ANN). Computer-assisted software that was originally developed to automatically detect metastatic hotspots and calculate BSI led to the commercial development of aBSI (EXINI Diagnostics AB, Lund, Sweden) [2]. The algorithm in aBSI was primarily trained on European patient datasets, and it has been adopted in Europe and North America. The automatic BSIs from the software has high reproducibility, reduced observer variability, and clinical value for prognostic assessments and monitoring treatment [3, 4]. Although the preselected hotspots require user confirmation before final reporting, both software programs have been applied in clinical practice.

A collaboration of Japanese nuclear medicine physicians and radiologists, EXINI Diagnostics, and PDRadiopharma Co., Ltd. (formerly, FUJIFILM RI Pharma) created BONENAVI, which is a computer-assisted diagnostic software for bone scintigraphy. This software was trained on a large dataset of Japanese patients, with specific optimization for patterns and disease prevalence among a Japanese population [3]. It has been extensively applied as a biomarker of the number of bone metastases [4]. The excellent diagnostic performance of BONENAVI for Japanese patients with prostate cancer and its prognostic utility has been validated [5–11]. A BSI derived from BONENAVI^1^ has since become the standard tool for automatic BSI calculation in Japan, and has been adopted in clinical practice, multicenter research studies and recent therapeutic interventions.

Although aBSI and BONENAVI share conceptual foundations, including hotspot detection, skeletal segmentation, and automated BSI computation, their training datasets, image processing strategies, and neural network model architecture differ [3, 4, 6]. These differences might result in systematic differences in BSI output, especially when metastatic disease is extensive.

Despite the widespread application of both software, BSIs calculated by them have never been directly compared using identical scan data. Such comparisons are essential to assess the cross-applicability of BSIs among software platforms and to enable meaningful interpretation and comparisons of global findings. The purpose of this study was to identify correlations, agreement, and potential systematic bias between BSIs from aBSI and BONENAVI in a patient cohort.

## Methods

### Patients

We retrospectively analyzed 339 bone images from 34 patients with prostate cancer and bone metastases (between 2012 and 2025). All patients were diagnosed and followed up at the Department of Urology, Kanazawa University Hospital. All patients had at least one documented metastasis to bone confirmed by X-ray computed tomography (CT) and/or magnetic resonance imaging. The Institutional Review Board at Kanazawa University approved this study (Approval No. 114831, 2024 − 379) and the requirement for informed consent was waived due to the retrospective design.

Sample size adequacy was evaluated using a paired equivalence test based on the Two One-Sided Tests (TOST) procedure after preliminary calculation of BSIs. With equivalence margins of ± 0.5 and ± 1.0 BSI, based on the observed mean difference (0.31) and standard deviation of paired differences (1.19), the required numbers of paired studies were estimated as 257 and 19, respectively. Thus, the 339 paired scan comparisons were sufficient for agreement analysis, although the repeated-measures structure across 34 patients should be considered.

## Imaging protocol

Metastatic bone disease in all patients was assessed using serial whole-body bone scintigraphy using standard clinical protocols. The patients were intravenously injected with 740 MBq of technetium-99m methylene diphosphonate (^99m^Tc-MDP; PDRadiopharma, Inc., Tokyo, Japan). Anterior and posterior whole-body images were acquired 3–4 h later using a dual-head gamma camera equipped with low-energy high-resolution collimators and the following single-photon emission computed tomography (SPECT)/CT instruments: Symbia T6, Symbia Intevo Bold, Symbia Intevo 16 (Siemens Healthineers, USA Inc., Hoffman Estates, IL, USA). The acquisition parameters of scan speed, 15 cm/min; matrix size, 256 × 1024, and energy window, 140 keV ± 7.5% followed standardized institutional protocols to ensure consistent examinations.

## Image data handling

All images were exported in Digital Imaging and Communications in Medicine (DICOM) format and transferred to dedicated nuclear medicine computers for automated analysis. Clinical information was collected from institutional medical records, and all imaging data were anonymized before analysis.

## Automated BSI analysis

Bone scan indexes were automatically calculated by aBSI v. 3.7.0. (EXINI Diagnostics, AB) and BONENAVI (v. 2.2. PDRadiopharma Co., Ltd.). A multiresolution registration model was used by aBSI to segment the skeleton and an iterative thresholding technique for hotspot detection. An ANN trained predominantly on European datasets was used to filter out pre-selected hotspots for physicians to review or edit [12]. BSI calculation was performed using cloud-based aBSI software accessed through a secure VPN connection. We also installed the desktop version of aBSI (EXINI bone) on a local computer and analyzed its performance with permission from EXINI diagnostics. Because the cloud-based aBSI and desktop EXINI bone share identical algorithms the term “aBSI” is used throughout this manuscript for simplicity. BONENAVI is based on the same principles for skeleton and hotspot segmentation but uses a different ANN-based architecture that was trained on large-scale Japanese multicenter bone scintigraphy datasets [3]. Although BONENAVI software was originally developed using this multicenter dataset, the patient cohort used in the present study was independent and did not include data from that original development dataset.

The BSIs derived from aBSI and BONENAVI (BSI_aBSI_ and BSI_BONENAVI_, respectively) use the same foundational image analysis of multiresolution registration model to segment the skeleton and an iterative thresholding technique to segment hotspots. Both use ANN-based algorithms to filter pre-selected hotspots. The aBSI has been further fine-tuned using simulated images to increase the linear response of BSIs to high disease burdens [6]. The ANN architecture has also been updated from a single level ensemble architecture in BONENAVI to a two-level sequential ANN architecture where the first model output feeds the second level ANN ensemble in aBSI.

The DICOM images were separately processed by aBSI and BONENAVI. Each automatically identified metastatic hotspots, segmented them into predefined skeletal regions, estimated the ratio (%) of skeletal involvement, and generated a BSIs (unit: % of total skeletal mass). No manual adjustment, lesion marking, or preprocessing was required before automated processing to ensure fair comparisons between the two types of software.

## Subgroup definition to analyze patients with high-BSI

We analyzed a predefined subgroup with BSI_BONENAVI_ > 7% as the reference software in Japan to determine discrepancies in patients with extensive bone metastasis. This group represented patients with substantial metastatic burdens that might cause automated hotspot detection and segmentation algorithms to behave differently. The threshold was selected based on the finding that inter-method variability increases with higher metastatic burdens [13].

### Relationship between aBSI and BONENAVI

We explored alternative regression models because the relationship between BSI_aBSI_ and BSI_BONENAVI_ was not strictly linear, particularly at higher BSIs. Models were fitted using Wolfram Language (Wolfram Research, Champaign, IL, USA). In addition to linear regression, a combined linear-exponential model was applied to capture systematic divergence at higher BSIs.

## BSI changes during follow-up studies

Longitudinal changes in BSIs (ΔBSIs) were calculated, and concordance between ΔBSI_BONENAVI_ and ΔBSI_aBSI_ was assessed using data from patients who had been serially followed up. Using ΔBSI thresholds of ± 0.3 and ± 0.5, we categorized the follow-up findings as improved, not changed, or progressed, then evaluated agreement between aBSI and BONENAVI. These thresholds were selected based on the distribution of ΔBSI_aBSI_ (median, 0.00; interquartile range [IQR], -0.50–0.33), ΔBSI_BONENAVI_ (median, -0.01; IQR -0.48–0.26). We also defined 0.3% change as clinically significant, or 10% change from baseline BSI for flare phenomena as described [14, 15].

### Statistical analysis

All data are expressed as means ± standard deviation (SD). Sample size adequacy was estimated using a normal-approximation formula for paired equivalence testing based on TOST procedure. Linear associations between BSI values derived from the two types of software were assessed using Pearson correlation coefficients. We used Bland–Altman analyses to determine agreement and identify systematic biases, by calculating mean differences and 95% confidence intervals (CI). Kappa coefficients of 0.41−0.60, 0.61−0.80, and 0.81-1.00 were respectively considered as moderate, substantial, and almost perfect agreement. Cramer’s V was calculated as the square root of χ² / (n × minimum (*r* − 1, c − 1)), where n is the total sample size, r and c represent numbers of rows and columns, respectively, and χ² is the Pearson chi-square statistic. A Cramer’s V > 0.5 was considered to indicate a strong association. Values with *p* < 0.05 were considered significant. All data were statistically analyzed using JMP 18 software (SAS Institute, Cary, NC, USA) and Wolfram Language (Wolfram Research Inc., Champaign, IL. USA).

## Results

### Patients

Table 1 summarizes the demographics and baseline characteristics of 34 patients who had been examined by 339 bone scintigraphic images (mean number of scans, 10.0 ± 6.8 per patient (range, 1–26). The mean age of patients at baseline was 69.9 ± 9.1 (48–89) years.

**Table 1:** Characteristics of patients

Characteristics	Values
Number of patients	34
Total bone scans (n)	339
Age at baseline (y)	69.9 ± 9.1 (48–89)
Number of studies per patient (range)	9.97 ± 6.77 (1–26)
Automated BSI	
Mean BSI_aBSI_, %	3.23 ± 3.47
Mean BSI_BONENAVI_, %	2.91 ± 2.84
Mean ΔBSI_aBSI_, %	0.005 ± 1.50
Mean ΔBSI_BONENAVI_, %	-0.002 ± 1.05

Data are shown as numbers or means ± SD with ranges. BSI_aBSI_, automated BSI calculated by aBSI; BSI_BONENAVI_, BSI calculated by BONENAVI; BSI, bone scan index; ΔBSI_aBSI_ and ΔBSI_BONENAVI_, difference from previous BSI_aBSI_ and BSI_BONENAVI_, respectively

### Automated BSI Analysis

**Fig. 1:**
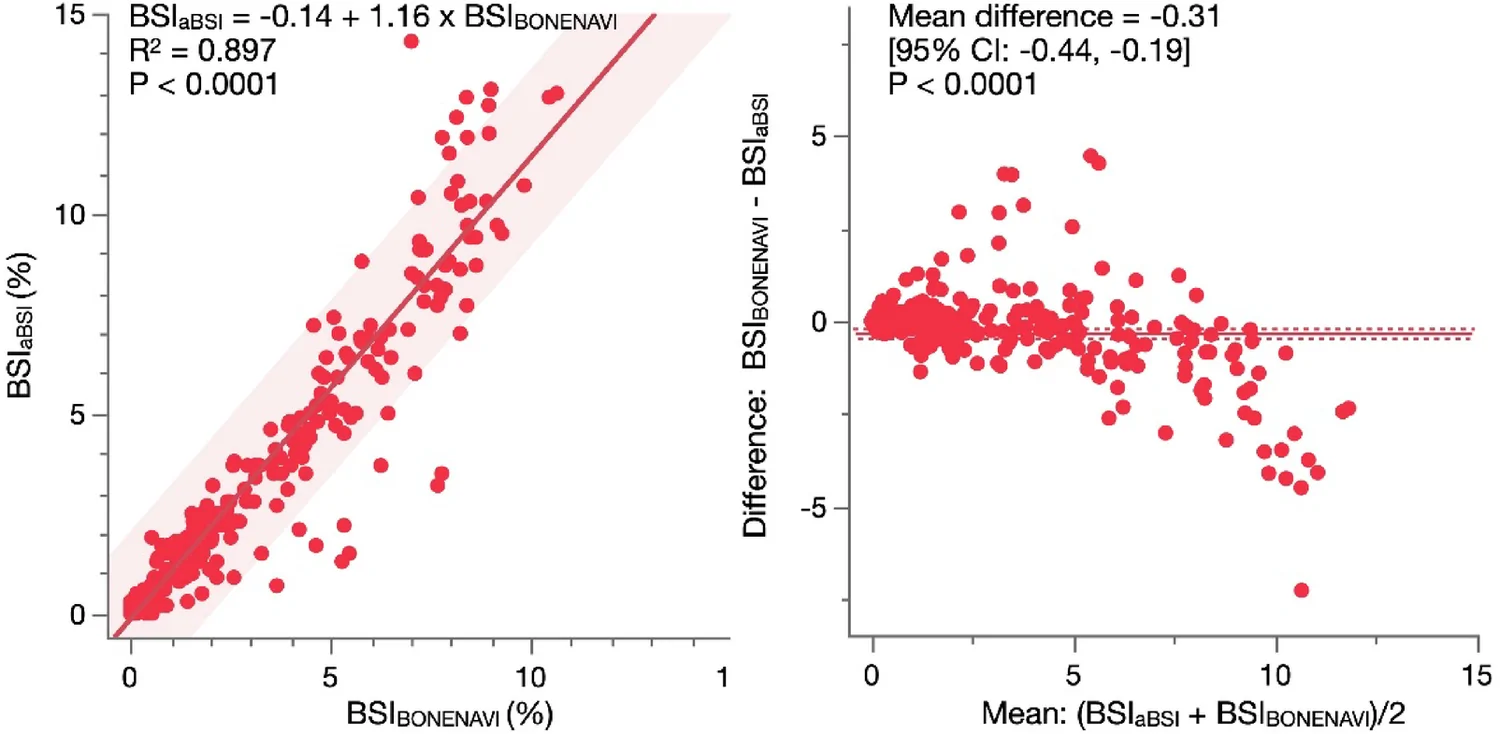
(**A**)Relationship between BSI_BONENAVI_and BSI_aBSI_ Solid line indicates regression line. Shaded area represents 95% CI. (**B**) Bland-Altman plot of BSI_BONENAVI_ and BSI_aBSI_. Solid line indicates mean difference, and dashed lines represent 95% CI. Mean difference in BSI measures was − 0.31% (95% CI, -0.44– -0.19; *p* < 0.001). Differences between BSI_aBSI_ and BSI_BONENAVI_ are relatively small and large when approximate mean BSIs are respectively < 7%, > 7%. BSI_aBSI_, automated BSI by aBSI; BSI_BONENAVI_, BSI by BONENAVI; BSI, bone scan index (%); CI, confidence interval

Correlations between BSIs derived from aBSI and BONENAVI were strongly positive. A correlation of *R*^*2*^= 0.897 (*p* < 0.001) indicated very strong associations. A scatter plot of BSI_aBSI_ vs. BSI_BONENAVI_ revealed their distribution close to the line of identity, particularly in the low-to-moderate BSI range (Fig. 1).

Despite this strong association, differences in the absolute BSI values produced by aBSI and BONENAVI were observed in the high BSI range. Bland–Altman analysis revealed a mean difference of -0.31% (*p* < 0.001), indicating that aBSI’s automatic BSI tended to be slightly higher than BONENAVI’s (Fig. 1, right). The 95% CI (-0.44%– -0.19%), suggested that while average bias was small, variability between each software increased in some images and became more apparent as BSI values increased.

### High-BSI subgroup

**Fig. 2:**
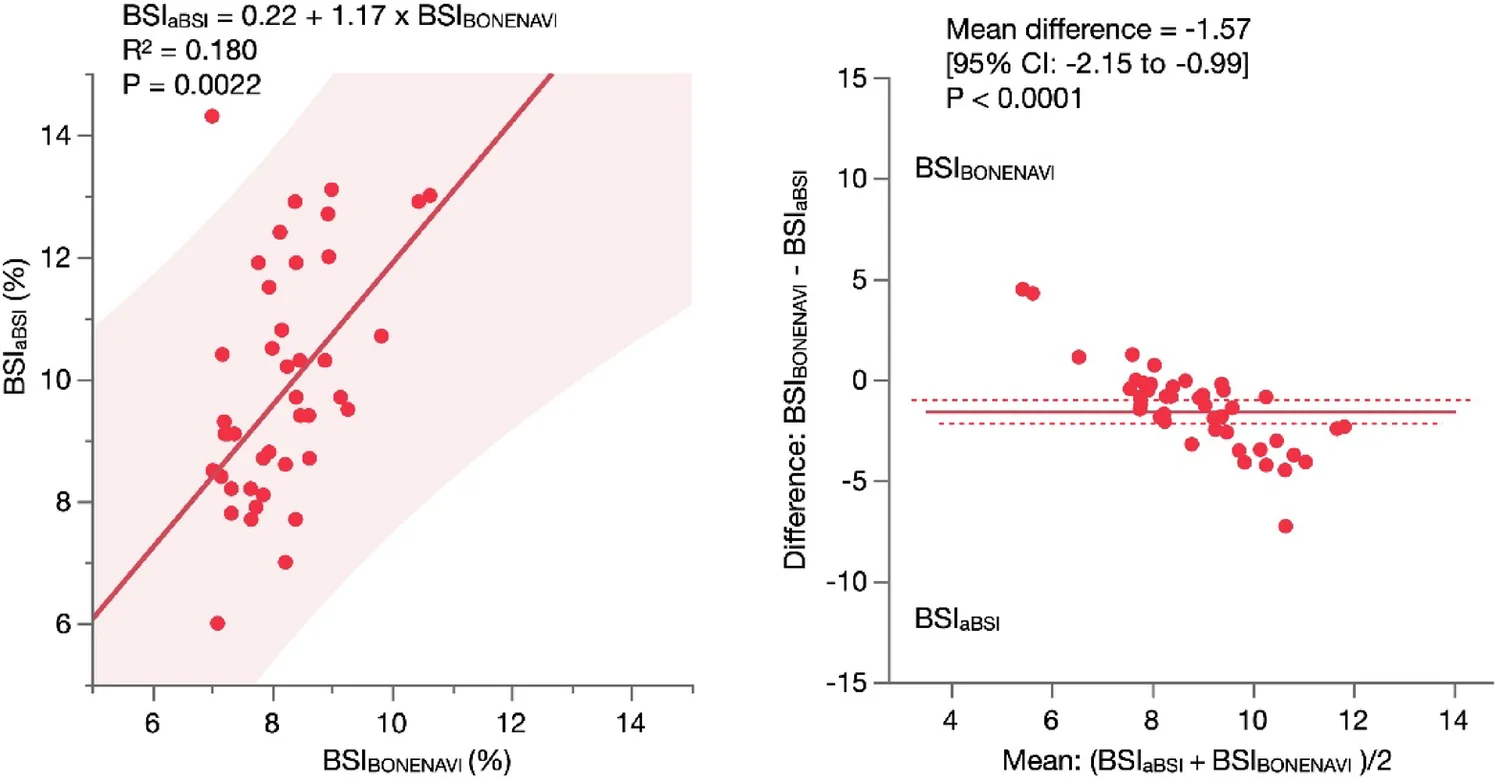
Results of subgroup analysis (**A**) Relationship between BSI_aBSI_ and BSI_BONENAVI_ with BSI_BONENAVI_ > 7. (**B**) Bland-Altman plot of BSI_aBSI_ and BSI_BONENAVI_ in subgroups. BSI_aBSI_, automated BSI by aBSI; BSI_BONENAVI_, BSI by BONENAVI; BSI, bone scan index (%)

The BSIs of 43 examinations of this high-BSI subgroup were all > 7.0%. Correlations between each software was reduced to *R*^*2*^ = 0.180 (*p* = 0.002) compared with the overall dataset (Fig. 2). This finding indicated only a weak-to-moderate association when patients had high burdens, suggesting that algorithms differed between the two automated BSIs.

**Fig. 3:**
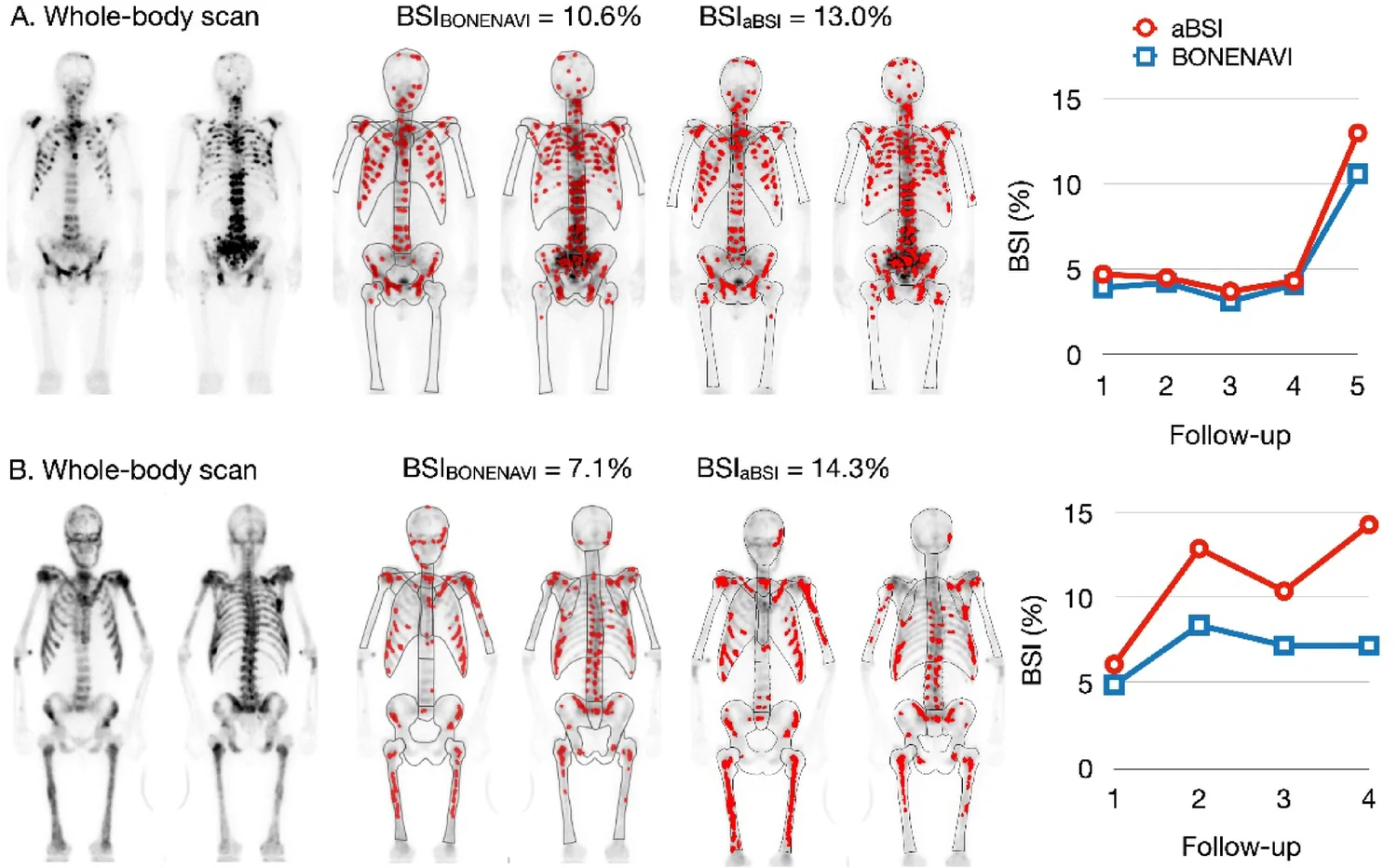
Representative cases illustrating concordant and discrepant BSI measurements between aBSI and BONENAVI. (**A**) Example with concordant longitudinal changes between the two software programs. (**B**) Example showing the largest inter-software discrepancy, with higher values obtained by aBSI. Whole-body scintigraphy and hotspot segmentation results are shown. The right panels demonstrate serial BSI changes during follow-up, with images corresponding to the highest BSI displayed. BSI_aBSI_, automated BSI by EXINI bone; BSI_BONENAVI_, BSI by BONENAVI; BSI, bone scan index (%)

The magnitude of inter-software differences was also substantially larger in this subgroup. The automatic BSIs calculated from images of patients with a high disease burden were higher for aBSI compared with BONENAVI, with a mean difference of + 1.57% (*p* < 0.001). The Bland–Altman results revealed a wide 95% CI range (0.99–2.15), which highlighted a pronounced increase in variability in patients with extensive metastatic disease (Fig. 2, right). Discrepancies exceeded 4%−5% in several individual evaluations, thus underscoring the potential clinical implications of software-specific differences in this subgroup. Figure 3 shows a patient with the greatest inter-software discrepancy.

### Best-fitted model of a relationship between aBSI and BONENAVI

**Fig. 4:**
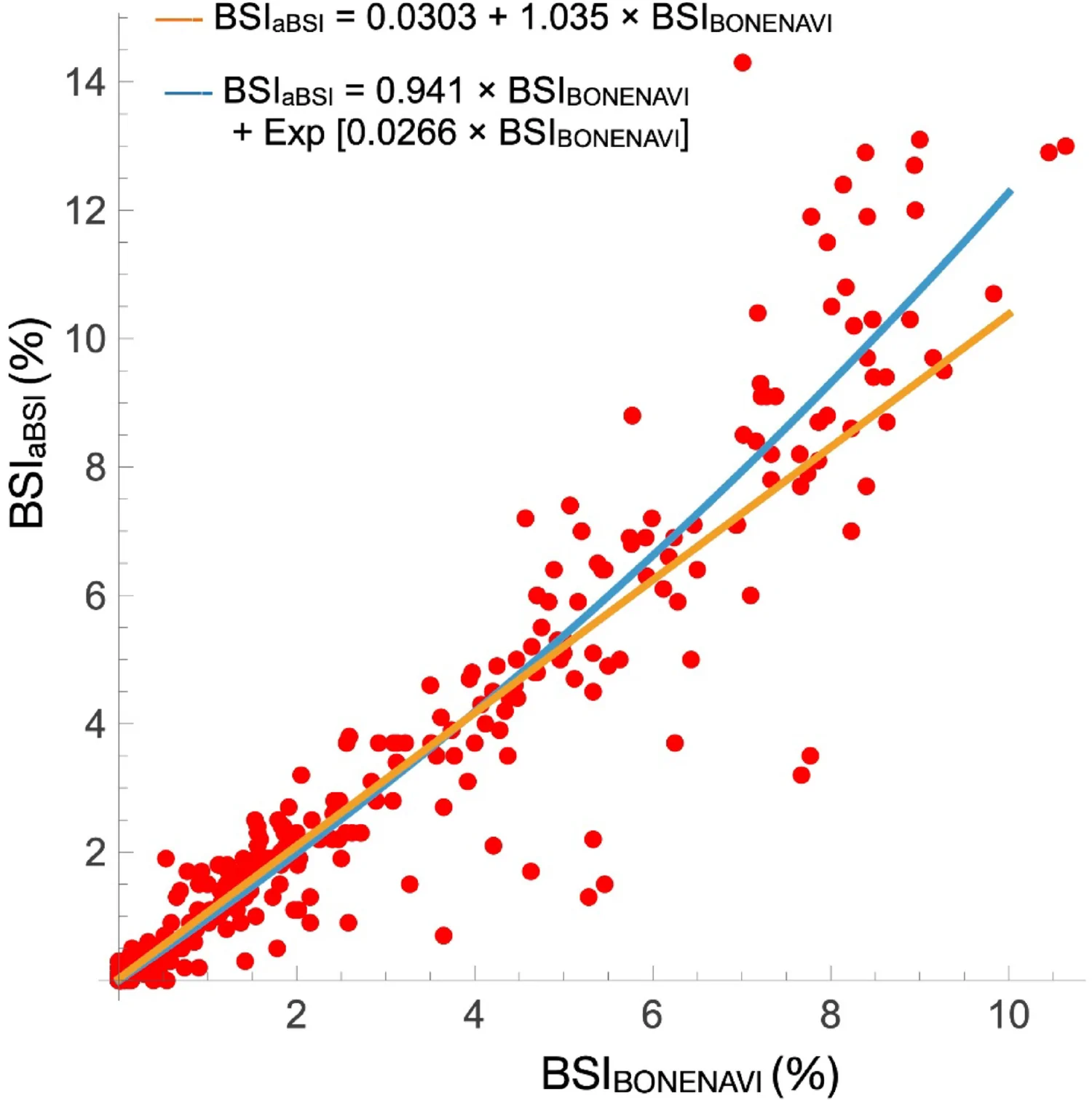
Linear (BSI_BONENAVI_ < 5) and non-linear plot of BSI_aBSI_ vs. BSI_BONENAVI_ BSI_aBSI_, automated BSI by aBSI; BSI_BONENAVI_, BSI by BONENAVI; BSI, bone scan index (%)

Scatter plots showed a non-linear relationship between BSI_aBSI_ and BSI_BONENAVI_. The two measures closely agreed at lower BSIs, whereas BSI_aBSI_ progressively exceeded BSI_BONENAVI_ of ~ 5% (Fig. 4). Consequently, a simple linear regression model derived from BSI_BONENAVI_ < 5% (BSI_aBSI_ = 0.0303 + 1.035 × BSI_BONENAVI_) data underestimated BSI_aBSI_ at higher BSIs. A combined linear and exponential model, BSI_aBSI_ = 0.941 × BSI_BONENAVI_ + Exp [0.0266 × BSI_BONENAVI_], provided a better representation of the relationship across the full range of BSIs, particularly in patients with higher metastatic burden.

### Changes in BSI

**Fig. 5:**
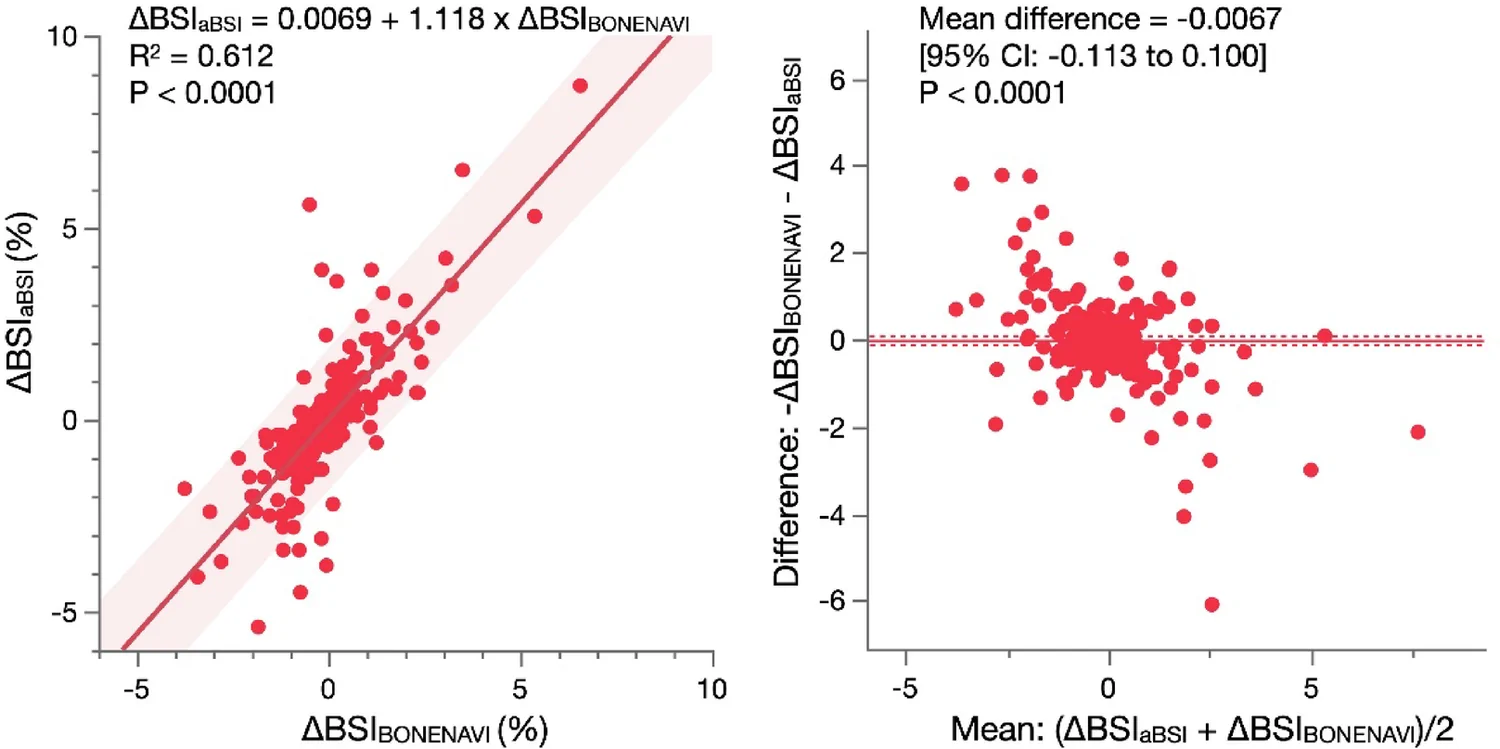
Changes and comparison of differences between ΔBSI_aBSI_ and ΔBSI_BONENAVI_ (**A**) Relationship between ΔBSI_aBSI_ and ΔBSI_BONENAVI_ from aBSI and BONENAVI, respectively. (B) Bland-Altman plot of differences between ΔBSI_BONENAVI_ and ΔBSI_aBSI_. aBSI, automated BSI by aBSI; BSI_BONENAVI_, BSI by BONENAVI; BSI, bone scan index (%)

The ΔBSIs between aBSI and BONENAVI significantly correlated. The results of a linear regression analysis in Fig. 5 show that ΔBSI_aBSI_ = 0.0069 + 1.118 × ΔBSI_BONENAVI_ (R^2^ = 0.612, *P* < 0.0001). When a ΔBSI threshold of ± 0.5% defined no change, the BSI classification changed to decreased, no change, and progression, indicating an overall agreement rate of 78.7% between the BSIs (Pearson χ^2^ = 259.9; *p* < 0.001), and κ = 0.63; 95% CI, 0.55−0.71; *p* < 0.0001) and Cramer’s V = 0.65, indicating a large effect size (Table 2). When the ΔBSI threshold was applied as ± 0.3, overall agreement was 76.4%, χ = 260.3, κ = 0.63; 95%CI, 0.56−0.71; *p* < 0.0001) and Cramer’s V = 0.65.

**Table 2:** Cross-tabulation of ΔBSI_aBSI_ and ΔBSI_BONENAVI_ (*n* = 305)

ΔBSI_BONENAVI_	ΔBSI_aBSI_		
	Decreased	Unchanged	Increased
Change rate ± 0.3%			
Decreased (< -0.3%)	71 (23.3%)	18 (5.9%)	2 (0.7%)
Unchanged (-0.3%–0.3%)	17 (5.6%)	107 (35.1%)	18 (5.9%)
Increased (> 0.3%)	2 (0.7%)	15 (4.9%)	55 (18.0%)
Change rate ± 0.5%			
Decreased (< -0.5%)	53 (17.4%)	16 (5.3%)	1 (0.3%)
Unchanged (-0.5%–0.5%)	17 (5.6%)	147 (48.2%)	21 (6.9%)
Increased (> 0.5%)	1 (0.3%)	9 (3.0%)	40 (13.1%)

ΔBSI_aBSI_ and ΔBSI_BONENAVI_, longitudinal changes of BSIs by aBSI and BONENAVI, respectively

## Discussion

We showed that although BSI values derived from BONENAVI and aBSI strongly correlated overall, clinically relevant discrepancies emerge in patients with a high bone metastatic burden. When the BSI_BONENAVI_ was > 7%, aBSI tended to yield significantly higher BSI values, with wide limits of agreement. These findings highlight important considerations regarding the use and interpretation of different automated BSI software programs, especially when patients have multiple bone metastases.

## Differences between BONENAVI and aBSI

A key factor underlying these discrepancies might relate to the development history and evolution of the automated BSI software. Differences between early aBSI releases and later revised versions have been directly assessed, and the results provide important context for interpreting our findings. The linearity and reproducibility of aBSI v. 2.0 is excellent across a BSI range up to 13% BSI (Pearson correlation *r* = 0.995) in phantom and validation settings [6]. The findings of upgraded version substantially mitigated several earlier concerns about the accuracy of higher BSIs.

Scatter plots revealed a non-linear relationship between BSI_aBSI_ and BSI_BONENAVI_, characterized by close agreement at lower BSIs and progressively higher BSI_aBSI_ when BSI_BONENAVI_ were 5%−6%. Accordingly, a simple linear regression model was insufficient to describe the relationship. We therefore applied a combined linear-exponential model, which improved representation of the data, particularly in the higher BSI range where divergence between the two types of software became evident. This behavior is consistent with increasing variance and systematic differences in hotspot detection and skeletal segmentation when the metastatic burden is high. The present proposed model is not intended for precise individual predictions, but it might facilitate approximate conversion between BSI_aBSI_ and BSI_BONENAVI_ when interpreting results from the different software programs.

BONENAVI was developed as an extension of an early version of EXINI bone, with modifications to better reflect characteristics of Japanese patients. When EXINI bone was tested on Japanese datasets, the sensitivity and specificity were 83% and 57%, respectively. The sensitivity and specificity of BONENAVI v. 1 with an incorporated Japanese database, were 90% and 81%, respectively when Japanese data were analyzed [16]. The BONENAVI version 1 was based on a database from one hospital, whereas version 2 was developed based on Japanese multi-center database that included numerous patients with various types of cancer. The diagnostic accuracy of version was improved, as the sensitivity and specificity were 90% and 84%, respectively, in men and 93% and 85%, respectively, in women [3]. Minor revisions for segmentation errors were applied and compared to previous versions, but the sensitivity and specificity did not significantly differ [17]. Neither of these reports specifically addressed consistency at high BSI levels, and BONENAVI has not undergone major revisions to date. Although BSIs > 15% have been found [12], such high BSIs are rare in Japanese clinical settings. BONENAVI was originally developed by adapting EXINI bone with a Japanese reference database. Therefore, its similar underlying algorithmic framework and reduced consistency at higher BSI levels might have contributed, at least in part, to the discrepancies found herein.

### Interpretation of changes in BSI

In this study, increased hot spots may have reflected not only metastatic lesions but also degenerative changes, treatment-related reactive changes associated with zoledronic acid or denosumab, and flare phenomena following therapy. However, in routine clinical practice, it is not always possible to definitively distinguish all hot spots as metastatic or benign. Baseline tumor burden and treatment responses to hormonal therapy, chemotherapy, and bone-modifying agents are all reflected in changes in BSI [5, 15]. Moreover, flare phenomena may appear as transient increases in tracer uptake caused by treatment-related reactive osteoblastic activity and are generally considered favorable findings after effective therapy [15]. Because the primary aim of this study was to compare changes in BSI values between the two software programs under real-world clinical conditions, such treatment-related changes were intentionally included rather than excluded.

Regarding the definition of significant change, there is no universally established threshold for BSI. A fixed absolute change (e.g., 0.3% or 0.5%) provides a simple and practical criterion, whereas relative changes (e.g., percentage change from baseline) may vary depending on the initial BSI value. For example, the same absolute change may have different clinical implications at low versus high baseline BSI levels. Although stratification according to baseline BSI could be considered, it would introduce additional complexity. We therefore adopted fixed thresholds (0.3% and 0.5%) for simplicity and consistency with previous reports, while acknowledging that interpretation of BSI changes in high-BSI cases may require additional caution.

### Regional differences in hot-spot detection

Regional differences in hot-spot detection may also have contributed to discrepancies between the two software programs. In some patients, divergence between BSI_aBSI_ and BSI_BONENAVI_ was more apparent in the upper extremities and femoral regions, whereas in other patients in whom BSI_aBSI_ was lower than BSI_BONENAVI_, discrepancies were more frequently observed in the vertebrae, sternum, and pelvis. These findings suggest that regional variation in lesion detection and classification may partially explain inter-software differences, particularly in patients with higher BSI values. However, the number of patients with marked discordance was limited in the present cohort, and the mechanisms underlying these regional variations remain unclear. Further investigation in a larger cohort, or in a dedicated study focusing on regional characteristics of lesion detection, would help clarify the factors contributing to these discrepancies.

## Limitations

This study has several limitations. First, we analyzed a cohort of patients with prostate cancer and bone metastases from a single center. Therefore, the generalizability of our findings might be limited. Statistical independence was not fully satisfied due to potential repeated-measures bias because we included serial examinations from the same patients. However, we compared the results generated by two software programs from identical images.

Second, multicenter validation studies are required to confirm whether inter-software differences are reproducible among diverse clinical settings. The threshold used for the subgroup analysis was empirically selected based on findings from our dataset rather than established clinical consensus or external validation. Although prior findings have suggested that the reproducibility of automated BSI measurements is reduced when patients have a very high metastatic burden, a universally accepted cutoff has not been established. Thus, our use of BSI_BONENAVI_ > 7% should be interpreted as an exploration intended to capture images with clearly extensive bone metastasis.

Finally, prognostic analysis was not performed because of the limited number of patients and follow-up data. Therefore, the prognostic implications of inter-software differences in BSI, particularly in patients with high BSI values, could not be evaluated. Because classification into progression, stability, or improvement did not differ substantially between methods, prognostic performance may be comparable; however, this hypothesis should be examined in future studies with larger cohorts and appropriate longitudinal follow-up.

## Conclusion

Our findings revealed that BSIs measured by two software programs correlated under most conditions. However, systematic differences were identified when patients had a high metastatic burden, which probably reflects algorithm design and training databases. Despite these discrepancies in absolute BSI values, longitudinal classifications of improvement, stability, and progression agreed between the two types of software supported cross-software and international comparisons of BSI measurements. Nevertheless, these differences underscore the need for careful interpretation in clinical practice and research, particularly in multicenter or international settings where various platforms and patient backgrounds differ.

## Data Availability

The datasets generated during and/or analyzed during the current study are available from the corresponding author on reasonable request.

## Abbreviations

ANN: Artificial neural network
BSI_BONENAVI_: Bone scan index by BONENAVI
BSI_aBSI_: Bone scan index by aBSI
BSI: Bone scan index
MDP: Methylene-diphosphonate

## Footnotes

1 BSIs derived from BONENAVI have also been described as aBSI in some Japanese studies [9, 10]. However, this usage should not be confused with “aBSI” software.
